# Large genomic rearrangements in the *CFTR *gene contribute to CBAVD

**DOI:** 10.1186/1471-2350-8-22

**Published:** 2007-04-20

**Authors:** Magali Taulan, Anne Girardet, Caroline Guittard, Jean-Pierre Altieri, Carine Templin, Christophe Beroud, Marie des Georges, Mireille Claustres

**Affiliations:** 1 INSERM, U 827, Montpellier, F-34000 France; 2 Université Montpellier1, UFR de Médecine, laboratoire de GénétiqueMoléculaire, Montpellier, F-3400 France; 3 CHU Montpellier, hôpital Arnaud de Villeneuve, laboratoire de Génétique Moléculaire, Montpellier, F-34000 France

## Abstract

**Background:**

By performing extensive scanning of whole coding and flanking sequences of the *CFTR (Cystic Fibrosis Transmembrane Conductance Regulator*) gene, we had previously identified point mutations in 167 out of 182 (91.7%) males with isolated congenital bilateral absence of the vas deferens (CBAVD). Conventional PCR-based methods of mutation analysis do not detect gross DNA lesions. In this study, we looked for large rearrangements within the whole *CFTR *locus in the 32 CBAVD patients with only one or no mutation.

**Methods:**

We developed a semi-quantitative fluorescent PCR assay (SQF-PCR), which relies on the comparison of the fluorescent profiles of multiplex PCR fragments obtained from different DNA samples. We confirmed the gross alterations by junction fragment amplification and identified their breakpoints by direct sequencing.

**Results:**

We detected two large genomic heterozygous deletions, one encompassing exon 2 (c.54-5811_c.164+2186del8108ins182) [or *CFTRdele2*], the other removing exons 22 to 24 (c.3964-3890_c.4443+3143del9454ins5) [or *CFTRdele 22_24*], in two males carrying a typical CBAVD mutation on the other parental *CFTR *allele. We present the first bioinformatic tool for exon phasing of the *CFTR *gene, which can help to rename the exons and the nomenclature of small mutations according to international recommendations and to predict the consequence of large rearrangements on the open reading frame.

**Conclusion:**

Identification of large rearrangements further expands the *CFTR *mutational spectrum in CBAVD and should now be systematically investigated. We have designed a simple test to specifically detect the presence or absence of the two rearrangements identified in this study.

## Background

Congenital bilateral absence of the vas deferens (CBAVD) accounts for approximately 3% of cases of male infertility in Caucasian populations. In about 85% of cases, CBAVD is recognized as an autosomal recessive disorder (MIM≠277180) associated with mutations in the cystic fibrosis transmembrane conductance regulator gene (*CFTR*, also symbolized ABCC7) [1-3]. Almost all of 1,500 different mutations so far identified in the *CFTR *gene in cystic fibrosis (CF) (MIM≠219700) or in related disorders (updates in the Cystic Fibrosis Mutation Database [4]) are single-nucleotide changes or small base pair(s) insertions or deletions in the exons or their flanking intronic sequences. The *CFTR *gene encodes a protein expressed in the apical membrane of exocrine epithelial cells which functions principally as a cAMP induced chloride channel and regulates also other ion channels. A combination of severe genetic changes in the two *CFTR *alleles that reduces *CFTR *function below 5% of physiological levels usually leads to the severe forms of classical CF [5,6]. Other so-called mild mutations that retain higher *CFTR *residual function can cause milder or incomplete phenotypes or *CFTR*-associated phenotypes. CBAVD is caused either by two mild mutations or by a severe and a mild mutation [3,7,8]. Routine testing for the most prevalent mutations in classical CF misses most *CFTR *gene alterations in the CBAVD phenotype, which can be detected only by scanning the 27 *CFTR *coding and flanking sequences [8,9]. However, despite exhaustive analysis of the *CFTR *gene, a proportion of mutations still remains unidentified in CBAVD, ranging from 15 to 40% depending both on the technologies used and the ethnicity of patients.

Recent studies in cases with classical cystic fibrosis suggested that gross rearrangements encompassing a single or several exons could account for a significant part of unidentified alleles. Large deletions have been discovered either fortuitously on the basis of uniparental inheritance of polymorphic markers [10-14] and Southern blotting [15] or through a systematic screening [16-19]. These mutations, when present at the heterozygous state, elude the PCR-based techniques designed to detect small DNA alterations.

In CBAVD, there is paucity of information about large genomic rearrangements and only two have been reported so far but without information on breakpoints sequences [17,20].

In this study, we used a semi-quantitative fluorescent multiplex PCR assay to screen samples from CBAVD patients that remained unresolved after extensive scanning of *CFTR *exons. Two large deletions have been identified, which further confirms the involvement of gross DNA alterations in CBAVD.

## Methods

### Nomenclature of mutations

We followed the international nomenclature guidelines recommended in the Human Genome Variation Society web page [21]. However, for convenience to readers used to the nomenclature of the Cystic Fibrosis Mutation Database [4], the usual names of mutations are also indicated [in italic and in brackets].

### Recruitment of CBAVD Samples with One or No CFTR mutation

Our current procedure to detect *CFTR *point mutations responsible for CBAVD includes the following steps: 1) the screening for 33 common CF mutations by using a commercial kit (CF OLA assay, Abbot, Rungis-France), 2) the scanning of all 27 exons and their intronic boundaries by using optimized laboratory DGGE *(Denaturing Gradient Gel Electrophoresis*) or dHPLC (*denaturing High Pressure Liquide phase Chromatography*) protocols, 3) the sequencing of any abnormal region to confirm and characterize the sequence change by using ABI Prism BigDye Terminator Cycle Sequencing kit (PE Applied Biosystems). In addition, we also screen DNAs for intronic splicing mutations c.1679+1634A>G [*1811+1.6KbA>G*] and c.3717+10kbC>T [*3849+10kbC>T*] using PRC-restriction with appropriate enzymes. The whole procedure has been previously applied to each of the 182 unrelated patients with documented CBAVD who have been referred to our lab so far, allowing us to detect one or two *CFTR *mutations in 167 of them [[8] and unpublished results]. Here we have further studied the 32 samples remaining with one (n = 17) or no (n = 15) mutation. Whenever possible, we studied the familial segregation of mutations and variations in order to determine whether sequence changes were located on a single or on the two parental *CFTR *alleles. Informed consent to *CFTR *studies had been previously obtained from patients and relatives at the time of referral.

### Search of Gross Rearrangements in the CFTR Gene by SQF-PCR Analysis

We used a semi-quantitative fluorescent multiplex PCR assay (SQF-PCR) developed in our laboratory for the detection of exon deletions and duplications in the *CFTR *gene. The method relies on the comparison of the fluorescent profiles of multiplex PCR fragments obtained from different samples, the amplification being stopped at the exponential phase. The 27 exons were grouped into three multiplex PCRs with one primer of each pair 5'-labeled with the 6-FAM fluorochrome (oligonucleotide primers available upon request). Amplification mixture included 5 to 20 pmol of each primer and 150 ng of genomic DNA in a 20 μl volume. Concentration and quality of DNAs were thoroughly determined prior amplification reactions. The PCR cycling conditions were selected using QIAGEN Multiplex PCR Kit according to the manufacturer's instructions (Qiagen, Courtaboeuf, France). Amplifications were performed with an initial denaturation step at 95°C for 15 min, followed by 20 cycles of 30 s at 95°C denaturation, 90 s at 57°C annealing and 1 min extension at 72°C, followed by a final extension at 72°C for 10 min, using a Gene Amp 9700 Thermal Cycler (Applied Biosystems, Foster City, CA). Amplified DNA fragments were separated on an ABI 310 or 3130xl sequencer at 60°C. Using Genescan and Genotyper (ABI 310) or GeneMapper (ABI 3130xl) softwares, the data were analyzed by superimposing fluorescent profiles of test and control DNAs and by calculating dosage quotient for area and height of all peaks [22] after normalization with an exon of the *DMD *gene.

### Confirmation of Deletions by Junction Fragment Amplification

We developed a simple and rapid test to confirm the identified deletions using two distinct duplex PCRs. For mutation c.54-5811_c.164+2186del8108ins182 [*CFTR*dele2], one pair of primers [23] amplifies wild type exon 2 while the other one has been designed to detect specifically the deleted allele [primers IVS1F (5'-TACACAAGGCTTGTCTTTAG-3') and IVS2R (5'GTTAAGCCAGATAATTCTGC-3' *(this study*)]. For mutation c.3964-3890_c.4443+3143del9454ins5 [*CFTR*dele22_24], we used one pair of primers to amplify wild type exon 22 [23] and primers IVS21-3940F and TAG+3303R [15] to detect the junction fragment of the deletion. PCR products were separated by electrophoresis on a 2% agarose gel and visualized by ethidium bromide staining. This test ensures an internal amplification control and can distinguish between homozygous and heterozygous samples for the deletion.

### Identification of Breakpoints

We characterized the two large rearrangements by sequencing the amplified fragments of genomic DNA encompassing the breakpoints using primers noted above.

### Computer-Assisted Sequence Analysis

Large portions of genomic DNA sequences within and around the rearrangements were searched for both low complexity/simple repeats and interspersed repeats using the *RepeatMasker *program and were analyzed for sequence similarity using the *BLAST *tool available through UCSC (University of California Santa Cruz) Genome Browser on Human (March 2006 assembly) [24]. To predict the effect of large deletions on the *CFTR *open reading frame sequences, we used the "exon-phasing tool" of the UMD^® ^software (Universal Mutation Database) [25,26], which gives access to a graphical presentation of all coding exons of a gene according to their phasing in order to predict, at least at the genomic level, the consequence of the deletion of one or more exons on the open reading frame of the *CFTR *gene.

## Results

Using a comprehensive *CFTR *gene analysis protocol we had previously identified *CFTR *mutations in 167/182 (91.7%) patients with documented CBAVD including 150 (82.4%) with two mutations and 17 (9.3%) with one mutation. In the current study we present the result of our investigations concerning the involvement of large rearrangements in the 32 remaining samples with one or no *CFTR *small mutation.

### Detection of two Complex CFTR Large Deletions and Confirmation of Deletion Breakpoint Junctions

**Figure 1:**
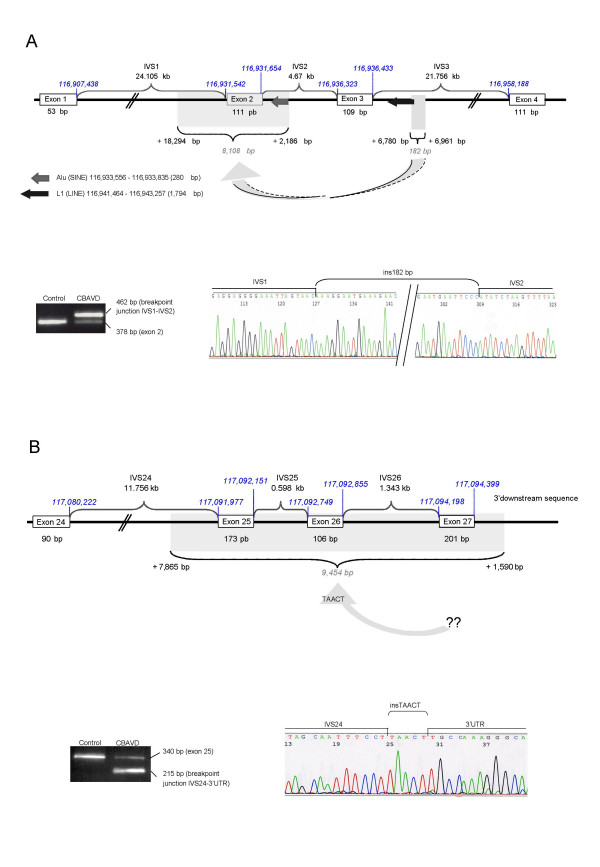
**A) Confirmation of complex deletion c.54-5811_c.164+2186del8108ins182 [*CFTR*dele2] in CBAVD**. Rearrangement c.54-5811_c.164+2186del8108ins182 (*upper panel*) consists of a gross deletion of 8108 bp spanning exon 2 (dotted area) and an insertion of 182 bp (grey area) at the deletion junction between the nucleotide 54-5811 of intron 1 (IVS1 nt 18294) and the nucleotide 164+2186 of intron 2 (IVS2 nt 2186) [according to the recommended international nomenclature with the A of the ATG start codon numbered as +1]. The 182-bp insertion is part of intron 3 between IVS3 nucleotides 6780 and 6961 but in inverted orientation (arrow). This complex in/del is also termed c.186-5811_c.296+2186del8108+ins182 with the A of the ATG translation start codon numbered as +133 in accordance with the GenBank reference sequence for the *CFTR *gene on chromosome 7 (NM_000492.2) and the CF mutation database [4]. It was recently reported as IVS1-5811_IVS2+2186del8108ins182 in one patient with cystic fibrosis [27]. A specifically-designed junction fragment amplification test (*lower panel*) confirmed the presence of the heterozygous deletion, indicated by specific PCR products on 2% agarose gels (*left*) by comparison with the non deleted allele. The deletion breakpoint junctions (indicated by vertical bars) and inserted sequences were determined by direct sequencing (*right*). **B) Confirmation of complex deletion c.3964-3890_c.4443+3143del9454ins5 [*CFTRdele22_24 *or *CFTR*dele25_27] in CBAVD**. Rearrangement c.3964-3890_c.4443+3143del9454ins5 (*upper panel*) consists of a gross deletion of 9454 bp (dotted area) encompassing exons 25 to 27 [*22 to 24*], the stop codon and the poly(A) signal. A small insertion of 5 bases (TAACT) was observed at the junction but is too small to allow any determination of its origin (arrow). The same rearrangement has recently been described in one patient with cystic fibrosis as c.4096-3890_c.4575+3143del9454ins5 with the A of the ATG translation start codon numbered as +133 in accordance with the GenBank reference sequence for the *CFTR *gene on chromosome 7 (NM_000492.2) or as IVS21-3890Stop+3143del9454insTAACT [15]. A specifically-designed junction fragment amplification test (*lower panel*) confirmed the presence of the heterozygous deletion, indicated by specific PCR products on 2% agarose gels (*left*) by comparison with the non deleted allele. The deletion breakpoint junctions (indicated by vertical bars) and inserted sequences were determined by direct sequencing (*right*).

Using SQF-PCR, a large genomic deletion was identified in 2 (6.25%) of the 32 samples analyzed in this study. Rearrangement c.54-5811_c.164+2186del8108ins182 [*CFTR*dele2] (Figure 1A) was found in a 27-year-old patient originating from Southern Italia carrying on the other allele the typical CBAVD splice variant IVS8(TG)12T(5). Rearrangement c.3964-3890_c.4443+3143del9454ins5 [*CFTR*dele22_24] (Figure 1B) was found in a 41-year-old man with Spanish and Sicily background carrying the missense mutation p.Arg170His. Both large deletions were found to be associated with a 7T allele at the IVS8(Tn) locus. The presence of either of the two deletions was confirmed by specific duplex PCRs amplifying both wild-type and deleted alleles in heterozygote samples. DNA sequencing showed that deletions breakpoint junctions appeared to be the same as those recently described in two patients with cystic fibrosis as IVS1-5811_IVS2+2186del8108ins182 [27] or IVS21-3890_Stop+3143del9454ins5 [15].

### Analysis of Genomic Sequences and Exon Phasing of the CFTR Gene

Despite the presence of Interspersed repeat elements in the vicinity of the rearrangements (Figure 1), there is no significant homology flanking the deletion breakpoints or insertion junctions to suggest that homologous recombination has occurred.

**Figure 2:**
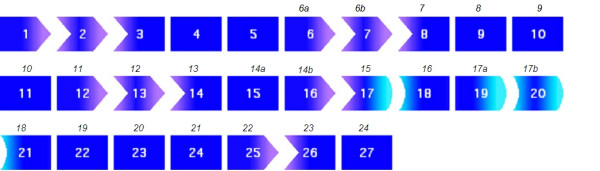
**Exon phasing of the *CFTR *gene (Universal Mutation Database [25])**. The *CFTR *gene comprises 27 exons which were previously numbered 1–24 [23] with subdivisions A and B for exons 6, 14 and 17, recognized as distinct units after the initial publication of the gene (GenBank NM_000492.2). Here we presents the numerotation of *CFTR *exons as described in UCSC (*University of California Santa Cruz*) Human Genome databases and updated as March 2006 Assembly [24]. Each exon is presented as a blue box with the international numbering within the box, and the familiar numbering above it. Each extremity of the box represents the specific phasing of the exon. *Left end of exons*: 1) vertical extremity: the exon begins by the first nucleotide of a codon; 2) curve: the exon begins by the second nucleotide of a codon; 3) arrow: the exon begins by the third nuceotide of a codon. *Right end of exons*: 1) vertical extremity: the exon ends by the last nucleotide of a codon; 2) light blue curve: the exon ends by the first nucleotide of a codon; 3) arrow: the exon ends by the second nucleotide of a codon. Large-scale deletions can introduce a translational frameshift (and lead to a premature termination codon) when two exons are joined end to end (for example, exons 1 and 4). When two exons in the same phase are joined, it is assumed that no frameshift occurs and that a shorter protein with an internal deletion may be produced.

The "exon-phasing tool" of the UMD^® ^software [25] uses a numerotation of *CFTR *exons in accordance with international nomenclatures and predicts an in-frame deletion of exon 2 (36 amino acids) for mutation c.54-5811_c.164+2186del8108ins182 [*CFTR*dele2] and the loss of the three last *CFTR *coding sequences (exons 22 to 24, that should be numbered 25 to 25, totalizing 159 amino acids) for mutation c.3964-3890_c.4443+3143del9454ins5 [*CFTR*dele22_24] (Figure 2).

## Discussion

Despite the accumulated information about genomic rearrangements in patients with cystic fibrosis, where they account for up to 16 to 24% of CF alleles negative for point mutations in European populations [15-18][27], only two large exonic rearrangements have been described so far in CBAVD [17,20]. The SQF-PCR protocol described in the present study enables the rapid and reliable detection of unknown *CFTR *gene deletions or duplications in CF or CBAVD. We identified two large rearrangements in two males carrying a typical CBAVD mutation on the other parental *CFTR *allele in a sample of 32 patients, which elucidates 4.25% of alleles that remained unidentified after standard investigations for point mutations. The remaining unsolved alleles might be explained by mutations in *CFTR *non-coding regions not explored by genomic DNA-based available methods or by the contribution of other(s) gene(s) to CBAVD. However if we postulate that, for most of the 15 patients with no mutation, CBAVD is probably not linked to *CFTR *defects, large rearrangements could represent 12% (2/17 alleles) of unidentified alleles, which is in the range of figures observed in the CF population. With this hypothesis, searching for large rearrangements in addition to conventional genotyping would identify two mutations in 152/167 (91%) and one mutation in 15/167 (9%) of CBAVD males. The two deletions found in our CBAVD samples are predicted to be "severe" alleles precluding the production of a functional *CFTR *protein and are thus null mutations. Bioinformatic analysis of the sequences flanking large rearrangements in the *CFTR *gene suggest that non-homologous end joining [28] is the most likely mechanism that is responsible for their occurrence, in accordance with recent studies [17,27].

## Conclusion

Screening for large genomic rearrangements in the *CFTR *gene is beneficial not only in classical cystic fibrosis but also in CBAVD, especially for those males who are carriers of a *CFTR *mild mutation. This study strengthens the importance to include screening of gross rearrangements in *CFTR *mutations scanning to offer adequate diagnosis and genetic counselling for CBAVD couples. International numerotation of exons should be used in order to avoid future misdiagnosis.

## Abbreviations

*CFTR*, cystic fibrosis transmembrane conductance regulator

CBAVD, congenital bilateral absence of the vas deferens

CF, cystic fibrosis

bp, base pair

PCR, polymerase chain reaction

SQF-PCR, semi-quantitative fluorescent PCR assay

## Competing interests

The author(s) declare that they have no competing interests.

## Authors' contributions

MT and AG wrote the manuscript; CG, JPA, CT performed the experimental work; CB designed the *CFTR *exon phasing tool and participated in the drawing of the figures. MdG and MC supervised the study and revised the manuscript. All authors read and approved the final manuscript.

## Pre-publication history

The pre-publication history for this paper can be accessed here:



## References

[B1] Anguiano A, Oates RD, Amos JA, Dean M, Gerrard B, Stewart C, Maher TA, White MB, Milunsky A (1992). Congenital bilateral absence of the vas deferens. A primarily genital form of cystic fibrosis. Jama.

[B2] Chillon M, Casals T, Mercier B, Bassas L, Lissens W, Silber S, Romey MC, Ruiz-Romero J, Verlingue C, Claustres M (1995). Mutations in the cystic fibrosis gene in patients with congenital absence of the vas deferens. N Engl J Med.

[B3] Claustres M (2005). Molecular pathology of the CFTR locus in male infertility. Reprod Biomed Online.

[B4] Cystic Fibrosis Mutation Database. http://www.genet.sickkids.on.ca/cftr/app.

[B5] Zielenski J (2000). Genotype and phenotype in cystic fibrosis. Respiration.

[B6] Mickle JE, Cutting GR (2000). Genotype-phenotype relationships in cystic fibrosis. Med Clin North Am.

[B7] Dork T, Dworniczak B, Aulehla-Scholz C, Wieczorek D, Bohm I, Mayerova A, Seydewitz HH, Nieschlag E, Meschede D, Horst J, Pander HJ, Sperling H, Ratjen F, Passarge E, Schmidtke J, Stuhrmann M (1997). Distinct spectrum of CFTR gene mutations in congenital absence of vas deferens. Hum Genet.

[B8] Claustres M, Guittard C, Bozon D, Chevalier F, Verlingue C, Ferec C, Girodon E, Cazeneuve C, Bienvenu T, Lalau G, Dumur V, Feldmann D, Bieth E, Blayau M, Clavel C, Creveaux I, Malinge MC, Monnier N, Malzac P, Mittre H, Chomel JC, Bonnefont JP, Iron A, Chery M, Georges MD (2000). Spectrum of CFTR mutations in cystic fibrosis and in congenital absence of the vas deferens in France. Hum Mutat.

[B9] Mak V, Zielenski J, Tsui LC, Durie P, Zini A, Martin S, Longley TB, Jarvi KA (1999). Proportion of cystic fibrosis gene mutations not detected by routine testing in men with obstructive azoospermia. Jama.

[B10] Morral N, Nunes V, Casals T, Cobos N, Asensio O, Dapena J, Estivill X (1993). Uniparental inheritance of microsatellite alleles of the cystic fibrosis gene (CFTR): identification of a 50 kilobase deletion. Hum Mol Genet.

[B11] Dork T, Macek M, Mekus F, Tummler B, Tzountzouris J, Casals T, Krebsova A, Koudova M, Sakmaryova I, Macek M, Vavrova V, Zemkova D, Ginter E, Petrova NV, Ivaschenko T, Baranov V, Witt M, Pogorzelski A, Bal J, Zekanowsky C, Wagner K, Stuhrmann M, Bauer I, Seydewitz HH, Neumann T, Jakubiczka S (2000). Characterization of a novel 21-kb deletion, CFTRdele2,3(21 kb), in the CFTR gene: a cystic fibrosis mutation of Slavic origin common in Central and East Europe. Hum Genet.

[B12] Costes B, Girodon E, Vidaud D, Flori E, Ardalan A, Conteville P, Fanen P, Niel F, Vidaud M, Goossens M (2000). Prenatal detection by real-time quantitative PCR and characterization of a new CFTR deletion, 3600+15kbdel5.3kb (or CFTRdele19). Clin Chem.

[B13] Chevalier-Porst F, Bonardot AM, Chazalette JP, Mathieu M, Bozon D (1998). 40 kilobase deletion (CF 40 kb del 4-10) removes exons 4 to 10 of the Cystic Fibrosis Transmembrane Conductance Regulator gene. Hum Mutat.

[B14] Lerer I, Laufer-Cahana A, Rivlin JR, Augarten A, Abeliovich D (1999). A large deletion mutation in the CFTR gene (3120+1Kbdel8.6Kb): a founder mutation in the Palestinian Arabs. Mutation in brief no. 231. Online. Hum Mutat.

[B15] Chevalier-Porst F, Souche G, Bozon D (2005). Identification and characterization of three large deletions and a deletion/polymorphism in the CFTR gene. Hum Mutat.

[B16] Audrezet MP, Chen JM, Raguenes O, Chuzhanova N, Giteau K, Le Marechal C, Quere I, Cooper DN, Ferec C (2004). Genomic rearrangements in the CFTR gene: extensive allelic heterogeneity and diverse mutational mechanisms. Hum Mutat.

[B17] Niel F, Martin J, Dastot-Le Moal F, Costes B, Boissier B, Delattre V, Goossens M, Girodon E (2004). Rapid detection of CFTR gene rearrangements impacts on genetic counselling in cystic fibrosis. J Med Genet.

[B18] Bombieri C, Bonizzato A, Castellani C, Assael BM, Pignatti PF (2005). Frequency of large CFTR gene rearrangements in Italian CF patients. Eur J Hum Genet.

[B19] Hantash FM, Redman JB, Starn K, Anderson B, Buller A, McGinniss MJ, Quan F, Peng M, Sun W, Strom CM (2006). Novel and recurrent rearrangements in the CFTR gene: clinical and laboratory implications for cystic fibrosis screening. Hum Genet.

[B20] Hantash FM, Milunsky A, Wang Z, Anderson B, Sun W, Anguiano A, Strom CM (2006). A large deletion in the CFTR gene in CBAVD. Genet Med.

[B21] Human Genome Variation Society web page. http://www.hgvs.org/mutnomen.

[B22] Yau SC, Bobrow M, Mathew CG, Abbs SJ (1996). Accurate diagnosis of carriers of deletions and duplications in Duchenne/Becker muscular dystrophy by fluorescent dosage analysis. J Med Genet.

[B23] Zielenski J, Rozmahel R, Bozon D, Kerem B, Grzelczak Z, Riordan JR, Rommens J, Tsui LC (1991). Genomic DNA sequence of the cystic fibrosis transmembrane conductance regulator (CFTR) gene. Genomics.

[B24] UCSC (University of California Santa Cruz) Human Genome databases. http://genome.ucsc.edu/.

[B25] Universal Mutation Database. http://www.umd.be.

[B26] Beroud C, Hamroun D, Collod-Beroud G, Boileau C, Soussi T, Claustres M (2005). UMD (Universal Mutation Database): 2005 update. Hum Mutat.

[B27] Ferec C, Casals T, Chuzhanova N, Macek M, Bienvenu T, Holubova A, King C, McDevitt T, Castellani C, Farrell PM, Sheridan M, Pantaleo SJ, Loumi O, Messaoud T, Cuppens H, Torricelli F, Cutting GR, Williamson R, Ramos MJ, Pignatti PF, Raguenes O, Cooper DN, Audrezet MP, Chen JM (2006). Gross genomic rearrangements involving deletions in the CFTR gene: characterization of six new events from a large cohort of hitherto unidentified cystic fibrosis chromosomes and meta-analysis of the underlying mechanisms. Eur J Hum Genet.

[B28] Lupski JR, Stankiewicz P (2005). Genomic disorders: molecular mechanisms for rearrangements and conveyed phenotypes. PLoS Genet.

